# The dynamics of actin protrusions can be controlled by tip-localized myosin motors

**DOI:** 10.1016/j.jbc.2023.105516

**Published:** 2023-11-30

**Authors:** Joseph A. Cirilo, Xiayi Liao, Benjamin J. Perrin, Christopher M. Yengo

**Affiliations:** 1 Department of Cellular and Molecular Physiology, Penn State College of Medicine, Hershey, Pennsylvania, USA; 2 Department of Biology, Indiana University – Purdue University, Indianapolis, Indiana, USA

**Keywords:** myosin, actin cytoskeleton, actin protrusion, filopodia, stereocilia, molecular motor, MYO3A

## Abstract

Class III myosins localize to inner ear hair cell stereocilia and are thought to be crucial for stereocilia length regulation. Mutations within the motor domain of MYO3A that disrupt its intrinsic motor properties have been associated with non-syndromic hearing loss, suggesting that the motor properties of MYO3A are critical for its function within stereocilia. In this study, we investigated the impact of a MYO3A hearing loss mutation, H442N, using both *in vitro* motor assays and cell biological studies. Our results demonstrate the mutation causes a dramatic increase in intrinsic motor properties, actin-activated ATPase and *in vitro* actin gliding velocity, as well as an increase in actin protrusion extension velocity. We propose that both “gain of function” and “loss of function” mutations in MYO3A can impair stereocilia length regulation, which is crucial for stereocilia formation during development and normal hearing. Furthermore, we generated chimeric MYO3A constructs that replace the MYO3A motor and neck domain with the motor and neck domain of other myosins. We found that duty ratio, fraction of ATPase cycle myosin is strongly bound to actin, is a critical motor property that dictates the ability to tip localize within filopodia. In addition, *in vitro* actin gliding velocities correlated extremely well with filopodial extension velocities over a wide range of gliding and extension velocities. Taken together, our data suggest a model in which tip-localized myosin motors exert force that slides the membrane tip-ward, which can combat membrane tension and enhance the actin polymerization rate that ultimately drives protrusion elongation.

Stereocilia are actin-based protrusions found in inner ear hair cells that function as mechanosensitive organelles crucial for the hearing and vestibular processes (1). The overall structure of stereocilia are similar to other actin-bundled protrusions such as filopodia and microvilli, with each containing a similar parallel actin-bundled core that is complemented by different actin binding proteins (2). Generally, the actin bundle of these protrusions is crosslinked by actin crosslinkers such as fascin and espin, which tightly pack the actin core and help regulate protrusion morphology. Unlike the dynamic filopodia and microvilli, stereocilia maintain their length and width throughout an organism’s life requiring precise control over their ultrastructure and dynamics (3).

The two vertebrate class III myosins, MYO3A and MYO3B, localize to the tips of stereocilia and have been implicated in stereocilia length regulation (4, 5, 6). The overall structure of MYO3A and MYO3B is similar, with both containing an N-terminal kinase domain followed by a canonical myosin motor and a neck region consisting of two calmodulin-binding IQ domains and an espin (ESPN-1/ESPN-L) binding domain in their tail region. However, the tail region of MYO3A is longer and contains a binding region for its binding partner MORN4, a third putative IQ motif, and a second tail homology domain (THDII) that binds actin and is required for MYO3A tip localization. Since MYO3B lacks this actin-binding domain, it is proposed to utilize the actin-binding domain of bound espin to provide a second actin contact point and be able to tip localize (7).

Both MYO3A and MYO3B have been proposed to be important for regulating stereocilia elongation during development, with mutations in MYO3A that alter this function ultimately leading to non-syndromic hearing loss (DFNB30) (5, 8, 9, 10, 11, 12). Previous work from our lab has highlighted how changes to the MYO3A motor domain can alter actin protrusion dynamics in cell-based systems. A MYO3B/3A fusion protein that replaces the MYO3A motor and neck with the MYO3B motor and neck (MYO3B.3Atail) was able to localize to the tips of filopodia in COS7 cells, though the amount of localized myosin, filopodia length, and filopodia density were all reduced (6). Similarly, we found that the loss-of-function deafness-associated mutation L697W reduced the motor function of MYO3A *in vitro*, as well as in COS7 cells (5, 13). Kinetic studies demonstrated a reduction in actin-activated ATPase and *in vitro* motility velocity, while COS7 cells transfected with MYO3A L697W produced filopodia of shorter lengths that extended at a slower rate than WT. While these studies emphasize that loss of MYO3A function can result in protrusion dysregulation, there has been no report of a gain-of-function deafness mutation in MYO3A that increases the motor properties or protrusion dynamics.

Interestingly, a MYO3A/3B double KO mouse model had stereocilia that were thin and grossly over elongated, despite MYO3A expression inducing elongation of filopodia in COS7 cells (14, 15). However, myosin 15 (MYO15) also localizes to the tips of stereocilia and may be important for length regulation (16, 17). In contrast to the MYO3A/B KO mice, MYO15 KO mouse models have stereocilia that are short, suggesting a model by which MYO3A and MYO15 coordinate to control stereocilia length. Furthermore, previous studies demonstrated that the L697W mutant can compete with WT MYO3A and alter elongation in filopodia, further emphasizing how the interplay between myosin motors can control protrusion dynamics (13).

Within actin protrusions, myosin tails generally contain cargo binding domains that associate with various binding partners to translocate them to the protrusion tips, as well as membrane binding motifs that either directly (TH1, or Tail Homology 1, domain of MYO1A) or indirectly (MyTH4-FERM, or myosin tail homology; band 4.1, ezrin, radixin, moesin, domains, of MYO10, 7A, and 15A) associate with the plasma membrane (18, 19, 20, 21, 22). A recent study determined that tethering myosin motors to the membrane was sufficient for protrusion elongation (23). To demonstrate this, the authors utilized an inducible membrane binding motif to tether various myosin motors to the membrane and demonstrated robust filopodia formation and extension in the presence of membrane-bound motor activity. Overall, this study suggests that myosin-mediated force at protrusion tips is sufficient to control protrusion formation.

In this current study, we aimed to understand how a MYO3A deafness-associated mutation, H442N, would impact its ability to regulate actin protrusions (Fig. 1*A*). This mutation was identified in a genetic screen of 216 randomly selected Japanese hearing loss patients (24). In this study, five alleles for H442N were detected and the authors determined the mutation to be likely damaging by analysis with the mutation impact prediction software PolyPhen2. However, this study did not elucidate any mechanism by which this mutation would affect MYO3A function and result in hearing loss. The mutation is located proximal to the P-loop, a conserved region of the myosin motor important in coordinating nucleotide binding (Fig. 1*A*) (25). By using complementary biochemical, biophysical, and cell-based experiments, we examined how this mutation impacted motor function both *in vitro* and within actin protrusions. In addition, to further investigate how myosin motors are fine-tuned for their role in length regulation, we generated chimeric myosins that contained various myosin motor domains fused to the MYO3A tail, which allowed us to investigate the impact of key motor properties (*e.g.*, motor speed and ATPase activity) on filopodia in COS7 cells (Fig. 1*B* and Table S1). The results of our studies led us to propose a model by which myosin motors at the tips of actin protrusions control protrusion dynamics *via* associating with the membrane and exerting a protruding force that combats membrane tension. Changes in the intrinsic motor properties of these myosins can disrupt this regulation and ultimately lead to over or underextended protrusions.

**Figure 1:**
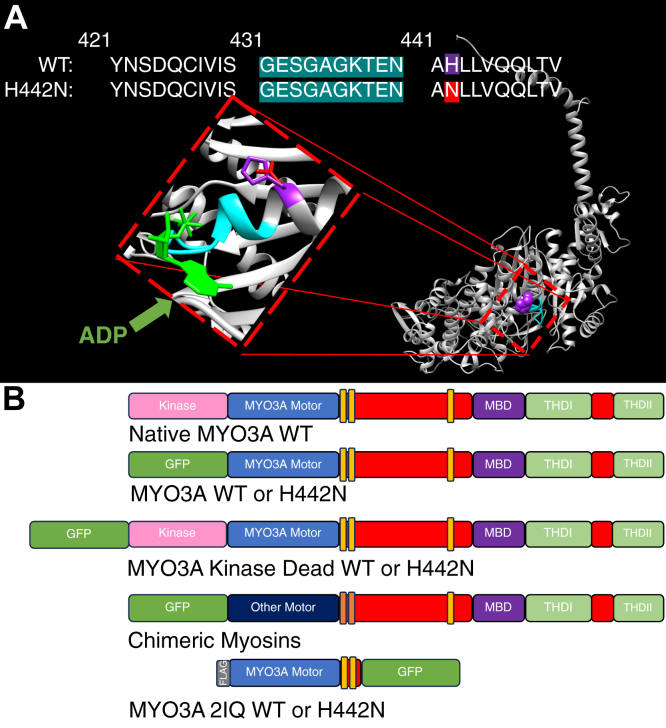
**Location of H442N mutation in MYO3A and diagram of MYO3A constructs.***A*, homology model of MYO3A 2IQ, lacking the kinase domain, generated using Chimera, highlighting the mutation H442N (original residue in *purple*, mutated residue in *red*) and its proximity to the P-Loop (*Blue*) and Nucleotide (*Green*). *B*, diagram of the domain structure of MYO3A WT and the various constructs used in this study.

## Results

We examined the biochemical and biophysical properties of the MYO3A motor by expressing and purifying a construct containing the motor domain and neck region but lacking the kinase domain, and containing a C-terminal GFP tag (MYO3A 2IQ; see structure and diagram in Fig. 1, *A* and *B*) (5, 6). To investigate the cellular function of MYO3A we expressed a full-length construct lacking the kinase domain and containing an N-terminal GFP tag (MYO3A; see Fig. 1*B*) (5, 6). We introduced the H442N mutation into both of these constructs which allowed us to determine how alterations in the *in vitro* biochemical/biophysical properties of the MYO3A motor alter its cellular function in actin protrusions. Finally, to examine the impact of the H442N mutation in stereocilia, we utilized a full-length MYO3A construct that contained the kinase domain but was mutated (K50R) to render it kinase-dead (MYO3A KD; see Fig. 1*B*) (15, 26).

To further investigate how myosin motor properties alter the dynamics of actin protrusions, we generated chimeric constructs containing the entire MYO3A tail but with the motor and first 2 IQ motifs of other myosin motors swapped in place of the MYO3A motor and first 2 IQ motifs (Fig. 1*B*, see Experimental procedures for additional information). Our approach was inspired by previous work from our group in which we swapped the MYO3B 2IQ region into MYO3A to create a MYO3B 2IQ.MYO3A tail chimera that successfully tip localized and elongated protrusions (7). In the current study, chimeras were generated by choosing myosins that were diverse in key motor properties. For example, myosins generate different speeds of movement depending on their ATPase cycle kinetics (27). In addition, the fraction of the ATPase cycle that myosin remains bound to actin filaments is referred to the duty ratio, and is an important determinant of whether a myosin can move processively along actin (*e.g.*, take multiply steps along actin without diffusing away) (28). The myosin motors we chose for our chimeras contained different motor speeds and duty ratios but were proven to be kinetically active *in vitro* with a similar neck length (Table S1). The different motor and 2IQ sequences were swapped into the MYO3A construct, which has proven to function inside COS7 cells despite the removal of its kinase domain. In addition, this allows for direct comparison between the MYO3A construct and chimeric myosins as each contains an N-terminal myosin motor domain, followed by two IQ motifs and the MYO3A tail domain. This allowed us to investigate how changing the motor properties, but not MYO3A tail interactions, affected MYO3A localization and protrusion dynamics.

### Impact of H442N on MYO3A ATPase and *in vitro* motility

**Figure 2:**
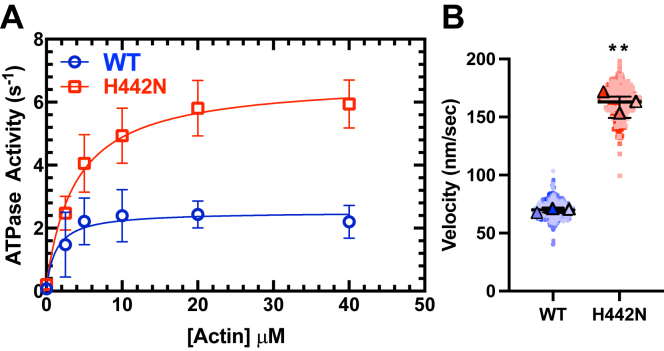
**Impact of H442N on intrinsic motor properties.***A*, the steady state actin-activated ATPase activity of MYO3A 2IQ (WT and H442N) was plotted as a function of actin concentration and the data fit to a hyperbolic function. The rate constants for basal ATPase activity (*v*_0_), maximal ATPase activity (*k*_cat_), and the actin concentration at which ATPase is one-half maximal (*K*_ATPase_) were determined (see Table 1). *B*, *in vitro* actin gliding velocities were measured in the *in vitro* motility assay for MYO3A 2IQ (WT and H442N). Data are from 3 experiments from three separate protein preparations (error bars are ±SD). *In vitro* motility data (100 filaments analyzed per experiment, for a total of 300 filaments per condition) are plotted as a Superplot (*t* test with Welch Correction, ∗∗ indicates *p* < 0.005).

The ATPase results for MYO3A 2IQ H442N demonstrated a ∼2.5-fold increase in maximal ATPase (*k*_cat_) as compared to WT (Fig. 2*A* and Table 1). The actin concentration at which the ATPase activity is one-half maximal (*K*_ATPase_) was not significantly different. We examined the motile properties of the MYO3A 2IQ constructs using the *in vitro* motility assay. We utilized a GFP antibody to attach the C-terminal end of the myosin to the motility surface (Movie S1). We found that the mutant MYO3A had a ∼2.5-fold increase in *in vitro* motility as compared to the WT (WT: 70.01 ± 2.03 nm/s, H442N: 163.40 ± 13.84 nm/s, *p* < 0.05) (Fig. 2*B* and Table 1). We also examined the *in vitro* motility as a function of the density of MYO3A 2IQ on the motility surface and found no significant difference between densities for WT and only a significant increase in motility at the 0.3 μM density for H442N (*p* < 0.005 (Fig. S1*A*). Overall, our results demonstrate that the mutation enhances the enzymatic and motile properties of MYO3A.

**Table 1:** Summary of rate constants from the ATPase and *in vitro* motility assays

MYOSIN	*v*_0_ (s^−1^)	*k*_cat_ (s^−1^)	*K*_ATPase_ (μM)	IVM velocity (nm/s)
MYO3A WT	0.07 ± 0.01	2.44 ± 0.30	1.30 ± 0.99	70.01 ± 2.03
MYO3A H442N	0.21 ± 0.01∗∗	6.50 ± 0.43∗∗	3.90 ± 0.96	163.40 ± 13.84∗∗

N = 3 separate experiments from three separate protein preps. *p*-values generated from a student’s *t* test. ∗∗ *p* < 0.005. Error bars for *k*_cat_ and *K*_ATPase_ are SE of the fit. Error bars for *v*_0_ and IVM Velocity are SD.

### Impact of H442N on MYO3A Subcellular localization and protrusion dynamics

**Figure 3:**
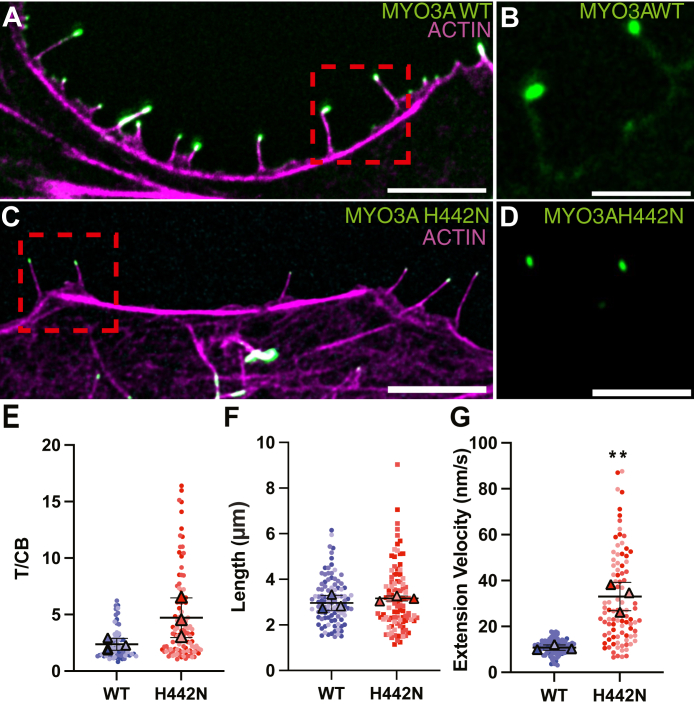
**Impact of H442N on cellular localization.** Confocal images of fixed COS7 cells transfected with WT (*A*) or H442N (*C*) MYO3A and stained with Alexa Flour 555 phalloidin (actin). Scale bars for (*A* and *C*) are 5 μm. *B*, and (*D*) are zoomed versions of the *red boxes* in (*A*) and (*C*), and are GFP-only images of representative filopodia from MYO3A WT or H442N, respectively, demonstrating the robust tip localization. Scale bars for (*B*) and (*D*) are 2.5 μm. *Green* is myosin and *pink* is actin. *E*, the ability to localize to the tips of filopodia was measured with a tip-to-cell body ratio (T/CB). *F*, lengths of filopodia protruding from the edge of COS7 cells. *G*, extension velocities of filopodia. N = 3 experiments for each measurement. Data are plotted as Superplots; ∗∗ indicates *p* < 0.005. Error bars are ±SD.

To investigate the impact of the H442N mutation on MYO3A localization and actin protrusion dynamics, we transfected COS7 cells with MYO3A WT or H442N for 24 h and imaged cells *via* both fixed cell confocal and live cell TIRF microscopy. COS7 cells, which readily produce filopodia upon transfection with MYO3A, are a useful model for assessing MYO3A cellular function. COS7 cells do not contain the known MYO3A binding partners ESPN and MORN4 allowing us to examine the impact of motor properties independent of binding partners (29). Furthermore, filopodia have an overall similar structure to stereocilia, with a tightly packed, parallel bundled actin core (2), affording the myosin a similar track to perform its cellular function. Each motor swap construct was transfected in parallel with MYO3A WT on three different days (N = 3 experiments) and no more than ten filopodia per cell, from at least ten different cells were measured. The number of filopodia per cell was limited to ten per cell to reduce oversampling from one cell, with a goal of reaching ∼100 filopodia from ∼10 cells per condition. The ten filopodia that were chosen could be tracked from initiation through full extension, with a final full extension of at least 1 μm in length. First, we observed robust localization of the myosin to filopodia tips for both WT and H442N (Fig. 3, *A*–*D*). We then measured tip-localization efficiency *via* tip-to-cell body ratio (T/CB) as previously described (5). We found that the T/CB ratio of H442N containing filopodia was unchanged from WT (Fig. 3*E* and Table 2) despite a broader distribution of T/CB ratios (SD = 0.52 and 1.76 for WT and H442N, respectively) (Table 2). We then measured filopodia length and found that MYO3A WT and H442N generated filopodia of similar length (Fig. 3*F* and Table 2). For live cell microscopy, we generated 20 min movies and tracked filopodia as they extended from the edge of the cell, until the conclusion of their extension (Movies S2 and S3). For both WT and H442N, we often saw myosin accumulation at the base of the protrusion that preceded the onset of extension, similar to what has been previously seen for MYO3A (6) (Movie S3). However, due to variability in the TIRF plane being imaged this accumulation was not observed for every measured extension. We measured filopodia extension velocities and found that filopodia containing MYO3A H442N had a 3-fold increased extension velocity as compared to WT (WT: 10.79 ± 1.14 nm/s, H442N: 33.05 ± 6.20 nm/s) (Fig. 3*G*, Table 2; Movies S2 and S3).

**Table 2:** Characteristics of filopodia containing MYO3A WT or H442N

MYOSIN	Extension velocity (nm/s)	Length (μm)	T/CB
MYO3A WT	10.79 ± 1.14	2.97 ± 0.33	2.37 ± 0.52
MYO3A H442N	33.05 ± 6.20∗∗	3.16 ± 0.11	4.72 ± 1.76

N = 3 separate experiments. *p*-values generated from a Welch’s *t* test. For T/CB, 69 filopodia from 10 cells were measured for WT; 100 filopodia from 10 cells were measured for H442N. For lengths, 100 filopodia from 10 cells were measured for each. For extensions, 100 filopodia from 10 cells were measured for WT and 93 filopodia from 10 cells were measured for H442N. ∗∗ *p* < 0.005. Error bars are ±SD.

### Stereocilia imaging

**Figure 4:**
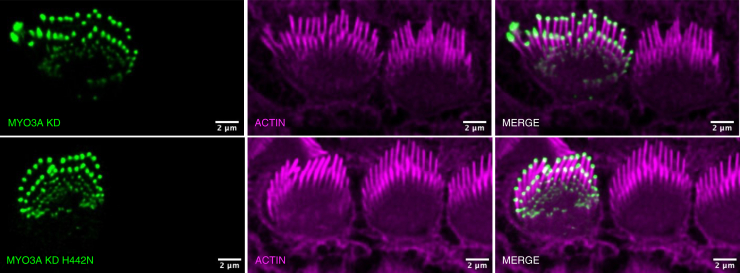
**Impact of H442N on localization to the tips of inner ear hair cell stereocilia.** Images of stereocilia of cultured hair cells from B6 P5 mice transfected with WT (*top*) or H442N (*bottom*) MYO3A Kinase Dead (KD) and stained with Alexa Flour 647 phalloidin (actin). *Green* is myosin and *pink* is actin. Transfected hair cells are shown on the *left* of each image, while un-transfected cells are shown on the *right* as a representative control.

To assess MYO3A localization in native stereocilia, inner ear hair cells were harvested from C57BL/6 (B6) P5 mice and cultured before being transfected with full-length MYO3A KD or MYO3A KD H442N. Cells were then fixed and stained with AlexaFluor 568 phalloidin (actin) and imaged *via* confocal microscopy. Both constructs were able to tip localize efficiently within stereocilia (Fig. 4). In this study, we did not examine changes in stereocilia length and morphology, since this would require additional experiments such as scanning electron microscopy.

### Motor-swapped chimeras: filopodia tip localization

**Figure 5:**
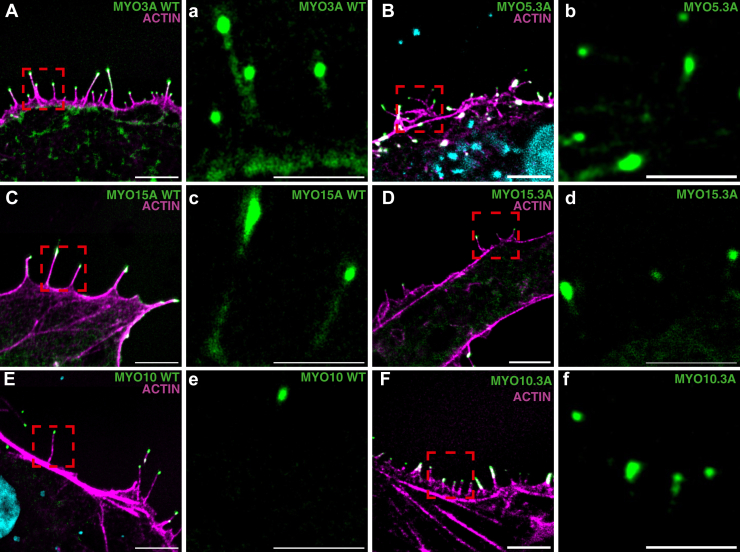
**Localization of chimeric myosin constructs in COS7 cells.** Representative confocal images of fixed COS7 cells transfected with WT MYO3A (*A*) or tip-localizing chimeric myosins (*B*–*F*) and stained with Alexa Flour 555 phalloidin (actin) and DAPI (nucleus). Scale bars for (*A*–*F*) are 5 μm. *a*–*f*, GFP only images of representative filopodia for each construct, demonstrating the robust tip localization. Scale bars for a-f are 2.5 μm. *Green* is myosin, *pink* is actin, and *blue* is DAPI.

To further investigate how the motor domain impacts myosin tip localization, COS7 cells were transfected with the various chimeric MYO3A constructs for 24 h, after which the cells were imaged *via* confocal microscopy. We examined motor-swapped MYO3A chimeras containing the motor and neck domain of MYO1A, MYH9 – (referred to as non-muscle myosin IIA, NMIIA), MYO5A, MYO7A, MYO10, and MYO15 (MYO1A.3A, NMIIA.3A, MYO5A.3A, MYO7A.3A, MYO10.3A, and MYO15.3A, respectively; Fig. 1*B*). WT MYO3A, MYO10, and MYO15, all of which are known tip localizing myosins, were also transfected as controls. While the WT myosins tip localized as expected, MYO5.3A, MYO10.3A, and MYO15.3A were the only chimeric myosins to tip localize (Fig. 5). The pattern of localization for the chimeric myosins was similar to MYO3A WT, with most of the myosin found at the filopodia tip (Fig. S3). However, in some images myosin was also found along the length of the filopodia (Figs. 5 and S3). COS7 cells transfected with MYO1.3A, MYO7A.3A, and NMIIA.3A did not produce many filopodia and there was no evidence of tip localization in the few filopodia produced. To ensure that the lack of tip localization was not due to an ability to initiate filopodia formation, chimeric myosins were also transfected into HeLa cells, which readily produce filopodia independent of myosin transfection. Upon transfection, we observed similar results whereby the chimeric MYO1A.3A, NMIIA.3A, and MYO7A.3A were unable to tip localize (Fig. S4).

### Motor-swapped chimeras: filopodia properties

**Figure 6:**
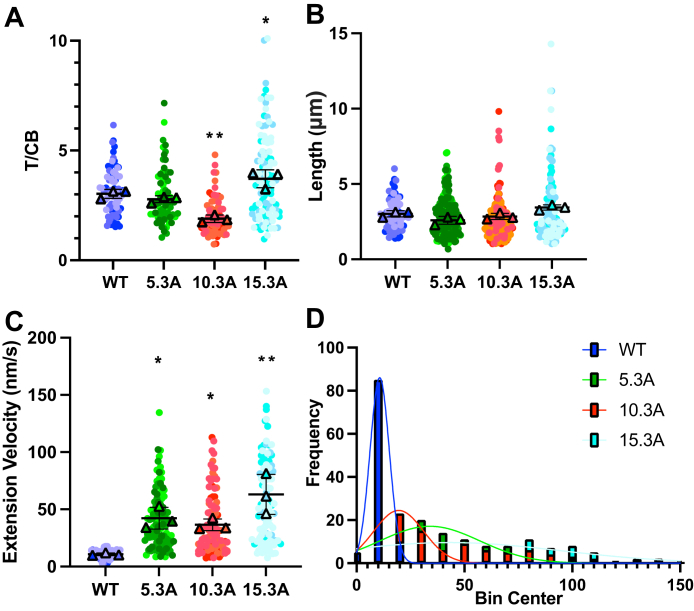
**Impact of chimeric myosin constructs on filopodia length and dynamics**. COS7 cells were transfected with WT or a chimeric MYO3A construct and various filopodia characteristics were measured. *A*, tip localization potential was examined by tip-to-cell body ratio (T/CB). *B*, lengths and (*C*) extension velocities of filopodia protruding from the edge of COS7 cells. *D*, histogram of extension velocity distributions for each construct. Data in (*A–C*) are plotted as SuperPlots. N = 3 experiments for each measurement. See Table 3 for a summary. ∗ *p* < 0.05, ∗∗ *p* < 0.005. Error bars are ±SD.

We also measured various properties of the filopodia that contained the tip-localizing chimeric myosins. Each construct was transfected on three different days (N = 3 experiments) and no more than ten filopodia per cell from at least ten different cells were measured to control for cell-to-cell variability. First, we measured tip-localization efficiency *via* tip-to-cell body ratio (T/CB) as previously described (14). We found that MYO10.3A localized less efficiently than WT MYO3A, while MYO15.3A localized more efficiently (Fig. 6*A*, and Table 3). We then measured the length of filopodia containing WT MYO3A or chimeric myosins. We determined there was no significant difference in protrusion length of filopodia containing each myosin (Fig. 6*B* and Table 3). Lastly, we measured extension velocity of filopodia containing WT MYO3A or chimeric myosins. We found that each of the chimeras extended at a velocity that was significantly increased as compared to WT MYO3A (Fig. 6*C*, Table 3, and Movies S1, S4, S5, and S6). Similarly, we observed a greater distribution in extension velocities for chimeric myosins as compared to WT MYO3A (velocities in nm/s; MYO3A: 10.79 ± 1.14, MYO5.3A: 36.53 ± 5.12, MYO10.3A: 42.30 ± 9.50, MYO15.3A: 63.10 ± 17.54, Fig. 6*D*).

**Table 3:** Characteristics of filopodia containing motor-swapped chimeric myosins

MYOSIN	Extension velocity (nm/s)	Length (μm)	T/CB
MYO3A WT	10.79 ± 1.14	3.03 ± 0.20	2.03 ± 0.20
5.3 A	36.53 ± 5.12∗	2.85 ± 0.20	2.78 ± 0.16
10.3 A	42.30 ± 9.50∗	2.59 ± 0.26	1.89 ± 0.17∗∗
15.3 A	63.10 ± 17.54∗∗	3.45 ± 0.17	3.71 ± 0.41∗

*p*-values were generated from Kruskal-Wallis analyses followed by Dunn’s multiple comparison (MYO3A WT as control). ∗*p* < 0.05, ∗∗*p* < 0.005. N = 3 experiments. For extension velocity, 100 filopodia from 10 cells were measured for each condition. For lengths, 75 filopodia from 10 cells were measured for WT, 191 filopodia from 20 cells were measured for MYO5.3A, 181 filopodia from 20 cells were measured for MYO10.3A, and 108 measurements from 11 cells were measured for MYO15.3A. For T/CB, 75 filopodia from 10 cells were measured for WT, 68 filopodia from 10 cells were measured for MYO5.3A, 68 filopodia from 10 cells were measured for MYO10.3A, and 103 measurements from 11 cells were measured for MYO15.3A. Error bars are ±SD.

### Correlation between *in vitro* motility and extension velocity

**Figure 7:**
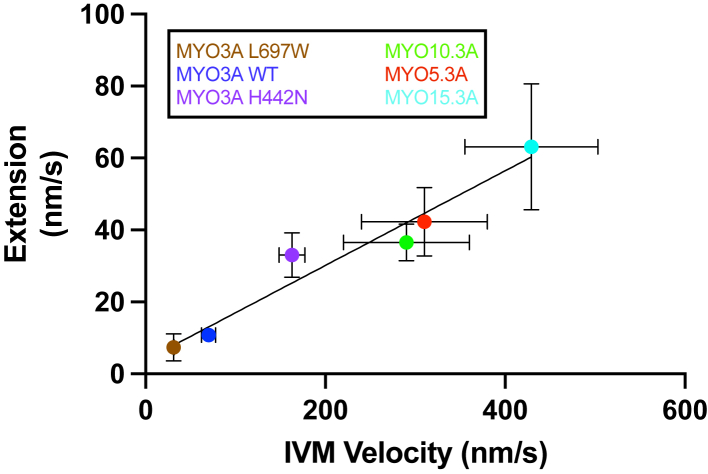
**Correlation between *in vitro* actin gliding velocity and filopodia extension velocity.** Linear regression of *in vitro* motility *versus* filopodia extension velocity for each construct examined. *In vitro* motility velocities are for myosin constructs of a similar neck length as measured by our lab (MYO3A WT and mutants) and other labs (MYO5A, MYO10, and MYO15 – see Table S1). Plotted extension velocities are measured for MYO3A chimeras. (R^2^ = 0.9492). Error bars are ±SD.

We then examined the relationship between *in vitro* motility and filopodia extension velocity. We plotted previously published *in vitro* motility velocities for each motor as a function of filopodia extension velocity. The results demonstrated a strong positive correlation between filopodia extension velocity and *in vitro* motility (Fig. 7, R^2^ = 0.9492). For this correlation, we specifically chose *in vitro* motility values calculated for constructs of the same/similar neck length (MYO10 3IQ, MYO15 2IQ, MYO5 2IQ) (30, 31, 32), since the length of the lever arm can greatly change the motile properties of myosin (33). Furthermore, since each construct contained the MYO3A WT tail domain, any interactions that the MYO3A tail may have within the cell (*i.e.*, membrane binding or protein-protein interactions) are kept consistent for each construct.

## Discussion

Our results provide important insight into the role of myosin motors localized to the tips of actin protrusions. We characterized a gain-of-function mutation in MYO3A, H442N, which is associated with hearing loss in a group of Japanese patients (24). Interestingly, this mutation enhanced the ATPase and motile properties of MYO3A, which correlated with its ability to increase the extension velocity of actin protrusions in a cultured cell line. Our results complement our previous work that found a loss-of-function deafness-associated mutation, L697W, depresses ATPase and motile properties while also causing a decrease in protrusion extensions in live cells. To further study this correlation between myosin motor properties and protrusion extension velocity, we generated a series of chimeras with the myosin motor domains swapped in place of the MYO3A motor. Our results clearly demonstrate a strong correlation between the *in vitro* actin gliding velocity and protrusion extension velocity over a wide range of velocities. Overall, our results suggest myosins are critical for controlling the growth of actin protrusions and provide fundamental information for understanding the biophysical properties of these crucial actin-based structures.

### Impact of H442N mutation on MYO3A

We observed a dramatic increase in the maximal actin-activated ATPase of H442N despite only a slight change in the apparent actin affinity (*K*_ATPase_) compared to WT. Thus, we can speculate on what steps in the ATPase cycle the mutant may alter. Since ATP hydrolysis was found to be rate-limiting in the kinase-removed construct (34), we predict hydrolysis is enhanced. We observed a ∼2.5-fold increase in actin gliding velocity for H442N compared to wild type. Our results suggest an increase in the ADP release rate constant in H442N could account for the enhanced actin gliding velocity since MYO3A was found to be detachment-limited in previous work (5, 13) and ADP release is typically the rate-limiting step for detachment (28, 35). Furthermore, there was no slowing of myosin motility at lower surface densities of myosin for either WT or H442N (Fig. S1), suggesting this change in velocity is independent of a change in duty ratio. We did observe higher velocities in the motility assay at the lowest surface density examined for H442N, which may suggest that H442N contributes higher drag forces at higher densities. Drag forces typically are caused by myosin heads that bind actin with higher affinity in the weakly bound states (36, 37). The duty ratio is an important determinant of the number of myosin motors in an ensemble that are engaged in motility and force generation (28). The proposed increase in hydrolysis may enhance entry into the strong actin-binding states while enhanced ADP release may accelerate entry into the weak binding states, and thus the overall change in duty ratio may be minimal. A homology model of the MYO3A motor suggests this mutation is located in close proximity to the P-loop of MYO3A (Fig. 1*A*), a conserved loop that is critical for coordinating the nucleotide triphosphate within the binding pocket of myosin (25). Members of the myosin super family have distinct profiles of P-loop conformations that are proposed to mediate nucleotide-favorable and unfavorable states (38). These structural states are generally predictive of the experimentally observed ADP-affinities and hence duty ratios, suggesting that P-loop orientation is critical for regulating intrinsic properties of the myosin motor. Given our results, it is possible that H442N alters the orientation of the P-loop such that it enhances dissociation of ADP from the binding pocket. Future studies will perform an in-depth kinetic analysis to understand how the mutation impacts ADP-release, as well as various other key steps in the ATPase cycle.

When transfected into COS7 cells, we observed robust tip localization of MYO3A H442N that was similar to WT MYO3A. The filopodia in the COS7 cells containing the mutant or WT MYO3A were of similar lengths, likely due to a maximum filopodia length phenomena discussed further below. However, we did observe an increase in filopodia extension velocity for protrusions that contained mutant MYO3A. These results correlate with the increase in *in vitro* motility velocity observed in the motility assay, as well as agree with our previous work on L697W (5, 13). Thus, we proposed that H442N is a gain-of-function mutation that increases the protrusion extension function and intrinsic motor properties of MYO3A. As such, these results demonstrate that both gain-of-function and loss-of-function mutations that alter intrinsic myosin motor properties can directly lead to defects in protrusion length regulation.

### Role of MYO3A in stereocilia

Our H442N results highlight a few possible roles for MYO3A within stereocilia. When expressed in B6 hair cells, both kinase-dead MYO3A WT and H442N localized similarly to the tips of stereocilia, suggesting that the mutation does not impair the ability of MYO3A to localize in its native environment. Given how both gain-of-function and loss-of-function mutations alter protrusion length regulation, MYO3A at stereocilia tips could exert a protrusive force that leads to extension during stereocilia formation. Indeed, mouse models found that MYO3A was critical during development, supporting this role for MYO3A (14). However, the protrusion elongation role for MYO3A is challenged by the MYO3A/B and MYO15 KO mouse models. In these mouse models, the MYO3A/B KO have longer and thinner stereocilia and the MYO15 KO have shorter stereocilia, suggesting MYO15 is the primary stereocilia elongator (14, 16). However, it is possible that MYO3A and MYO15 coordinate to elongate stereocilia through an unknown mechanism. For example, MYO15 may be primarily responsible for elongating, while MYO3A/B prevents over-elongation. One possibility is that both MYO15 and MYO3A produce a protruding force at stereocilia tips, however, since MYO3A is the slower motor, it gets pulled into a negatively strained conformation and thus functions as a brake, overall slowing and preventing over-elongation. Future studies investigating this coordination may shed light on novel mechanisms by which myosins can elongate protrusions.

Recent work by Moreland *et al.* (39) has highlighted that MYO15 can nucleate actin filaments and suggests a mechanism by which this nucleation can contribute to protrusion elongation. This proposed mechanism builds on earlier work which demonstrated skeletal muscle myosin can mediate nucleation of actin filaments, suggesting the potential for other myosins to also directly affect filament nucleation (40, 41). Thus, tip-localized myosin motors may impact actin protrusion dynamics by altering the actin polymerization mechanism both directly or indirectly, which will likely be an important aspect of future studies.

### Impact of motor properties on myosin localization

Our results suggest that the intrinsic motor properties of myosins can impact their ability to localize to the tips of protrusions. Myosins that form filaments and work as an ensemble to generate force, such as those in a muscle fiber, generally are fast motors that associate with actin for only a short fraction of their ATPase cycle (low duty ratio) (27, 28). Conversely, myosins that translocate along actin filaments to transport cargo as a single molecule, generally are slower and spend a larger fraction of their ATPase cycle bound to actin (high duty ratio). The chimeric myosins MYO1A.3A, NMIIA.3A, and MYO7A.3A all failed to localize to the tips of filopodia in both COS7 and HeLa cells (Fig. S4). For MYO1A and NMIIA, we propose that duty ratio limits these motors from being able to walk to the tips as they are low duty ratio motors (<0.1 and 0.05, respectively) and likely would be unable to stay attached to actin long enough to move forward before diffusing away (42, 43). Both the WT MYO7A and chimera MYO7A.3A failed to tip localize in COS7 cells, despite being a high duty ratio motor (0.60) (44). However, our MYO7A chimeric construct, which is predicted to translocate along actin quite slowly (2.7 nm/s), may be unable to walk faster than actin retrograde flow and tip localize (44, 45). Furthermore, a study by Fitz *et. al.* (23) found that the MYO5B motor was unable to induce protrusion formation and, therefore, unable to tip localize, despite having a duty ratio of ∼0.6 and sliding velocity of 381 nm/s (46). Therefore, other factors, such as mechanosensitivity, may also be important for determining tip localization efficiency (47). Overall, our findings suggest that intrinsic motor properties such as the duty ratio and motor walking speed of myosin motors are important in determining a motors ability to localize to the tips of actin-based protrusions (30, 42, 48, 49, 50, 51) (Table S1). Furthermore, our work suggests the H442N mutation does not reduce MYO3A duty ratio, since MYO3A H442N still tip localized in stereocilia and did not alter MYO3A tip localization in filopodia. Lastly, for both H442N and the chimeric myosins that did tip localize, we observed no difference in T/CB ratio. This is possibly due to a limited number of myosin binding sites on the plus end of the actin filaments that get saturated regardless of motor speed.

### Impact of motor properties on protrusion extension

We observed no difference in the length of protrusions containing either MYO3A WT or H442N, despite H442N having enhanced motor properties. Similarly, we observed no change in filopodia length between motor-swapped constructs when compared to WT MYO3A. Biophysical models of actin protrusions suggest there is a critical length that filopodia cannot extend beyond before buckling even if there is an increase in protruding force against the membrane (52). Our data support these models as we observed filopodia reaching this critical length and no longer extending.

We observed an increase in extension velocity for the motor-swapped myosins that correlated with the *in vitro* actin gliding velocity of the respective myosin motor. In the *in vitro* motility assay, an ensemble of myosins on the motility surface work together to glide actin. We propose a similar mechanism at protrusion tips, whereby an ensemble of myosins work together to exert a force that contributes to protrusion extension. In addition, since the number of motors at the tip does not change, we suggest the ensemble force is similar between constructs and further emphasize that the actin gliding velocity is an important determinant of protrusion extension velocity.

Our results suggest that the actin gliding velocity of tip-localized motors may be fine-tuned for controlled elongation of actin protrusions. For example, protrusions such as filopodia rapidly extend in order to properly contribute to cell motility, invasion, and endocytosis and thus require a faster motor like MYO10 (18, 53, 54). In contrast, stable protrusions such as stereocilia require a slower, controlled extension to allow for the complex ultrastructure to properly assemble during development, as well as maintenance and repair (22). Without this control (*i.e.*, in the case of mutations that alter these properties), the stereocilia may not properly assemble, resulting in changes in morphology that ultimately lead to hearing loss.

### Key factors important for actin protrusion dynamics

A recent study by Fitz *et al.* (23) determined that tethering myosin motors to the membrane was sufficient for protrusion elongation. The authors utilized an inducible membrane binding motif to tether various myosin motors to the membrane and demonstrated robust filopodia formation and extension in the presence of membrane-bound motor activity. Interestingly, the filopodia generated by this method reached a plateau length that was similar for each myosin motor, although the time it took to reach that plateau varied. These results are in good agreement with our work which demonstrates that each myosin motor generated protrusions of similar length, though at different rates of extension. Fitz *et al.* (23) also demonstrated that protrusion extension was limited by not only the amount of actin monomers available but also by the amount of membrane lipids. Lastly, the authors demonstrated that myosin-mediated protrusion extension was independent of VASP and formin, suggesting that these aspects of the canonical actin polymerization machinery are not necessarily limiting factors for protrusion extension. Thus, the Fitz *et al.* (23) study as well as our current study emphasizes the importance of several key factors in mediating protrusion elongation: (1) the availability of membrane lipids, (2) the availability of actin monomers, and (3) the myosin-based force generation at the protrusion tips.

Since direct membrane binding measurements with MYO3A are currently lacking, we can only speculate on if or how the tail domain interacts with the membrane. The MYO3A tail contains a large intrinsically disordered region which makes many biophysical studies of the tail difficult to perform. MORN4, the MYO3A tail binding partner, was originally thought to provide a link to the membrane but preliminary studies found that MORN4 has little or no membrane binding capacity (55). Thus, MYO3A membrane binding may be more complex and require the identification of novel binding partners as well as a detailed characterization of the tail domain.

### Model of actin protrusion elongation

**Figure 8:**
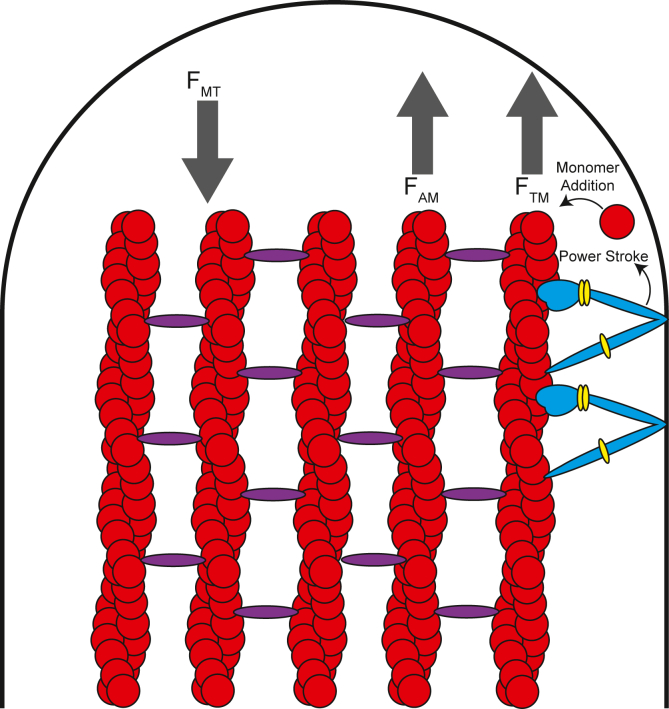
**Model describing the role of myosins in actin protrusions.** We propose that tip-localizing myosins translocate along actin filaments to the plus-ended protrusion tips. Tip-localized myosins associate with the membrane (*via* direct or indirect interactions) and undergo a tip-ward power stroke, generating force (F_AM_). The myosin tip-ward force, along with the force of actin polymerizing (F_A_) can push the membrane forward to combat membrane tension (F_MT_) and drive protrusion elongation. Furthermore, as the myosin glides actin forward, space is created between the membrane and the plus ends of the filaments, allowing for more actin monomer addition to occur. Changes in intrinsic motor properties due to either gain-of-function or loss-of-function mutations may disrupt this process, leading to impaired protrusion length/dynamics.

Overall, we propose that tip-localized myosins can exert a tipward force that not only relieves membrane tension but also acts similar to a Brownian ratchet to glide the membrane tipward and create space locally for more actin monomers to diffuse into the protrusion tip (Fig. 8) (56). From our data, we hypothesize that the faster motors accomplish this more rapidly, overall leading to faster rates of protrusion extension. Indeed, myosins associated with the membrane have been implicated in relieving membrane tension by directly applying a protrusive force on the membrane, examined both experimentally and with biophysical modeling (23, 52, 57). One biophysical model suggested that filopodia length is largely limited by the ability of actin polymerization forces to overcome the force of membrane tension, estimated to be about 10 to 20 pN given a radius of 50 to 100 nm (58). Indeed, another study by Kovar and Pollard identified that a single actin filament could produce force against the membrane ranging from 0.25 to 0.56 pN (59). Considering filopodia are estimated to contain 10 to 30 cross-linked actin filaments (60), and assuming all filament tips are in direct contact with the membrane at the filopodial tip, actin polymerization forces could contribute anywhere between 2.5 to 16.8 pN of force to drive protrusion. This model is consistent with experimental data that demonstrated that changes in rates of actin assembly can directly alter the dynamics of protrusions (61). However, as the filopodia extends and membrane tension begins to reach the upper limits (*i.e.*, ∼18–20 pN), the force of actin polymerization alone would not be sufficient to continue extension. Myosin motors accumulated at the protrusion tip could contribute to combatting this force, as well as create space for polymerization to occur. Recent data published by Shangguan and Rock (62) estimates ∼360 MYO10 molecules occupy a single filopodium, distributed mostly at the protrusion tip (62). Given that a single molecule of MYO10 can generate a maximum force of ∼1pN per single step (31) with a duty ratio of roughly 60% (48), an ensemble of ∼100 active MYO10 molecules at the protrusion tip could contribute around 60pN of tip-ward force along the membrane per step. The strong correlation between unloaded sliding velocity and extension velocity suggests that the tip localized myosin may function under sub-maximal loads. This assumption appears to be reasonable, given the number of myosin motors predicted to be localized to the tips and the amount of protrusive force required to overcome membrane tension.

## Conclusions

In this study, we characterized a naturally occurring deafness-associated mutation in MYO3A to gain insight into how alterations in MYO3A motor function can impact protrusion dynamics. Much like cardiomyopathy mutations in beta-cardiac myosin which can be gain or loss of function, mutations in MYO3A can alter motor activity in either direction, leading to changes in its ability to control protrusion length and elongation. Our results were complemented with chimeric myosin constructs that demonstrated a strong correlation between motor activity and extension velocity that is observed over a wide range of motor gliding and extension velocities. Based on our work and that of others in the field, we propose a simplified model of how myosin-based protrusive force influences elongation. However, we recognize that many other factors are likely to influence protrusion dynamics such as cargo transport, actin regulatory proteins, and membrane dynamics. Future studies will continue to investigate how the biophysical and biochemical properties of myosins localized to actin protrusions influence the length, width, and dynamics of these important actin-based structures, which play a critical role in hearing, balance, cancer metastasis, and wound healing.

## Experimental procedures

### Reagents

ATP was prepared from powder (Millipore-Sigma) and the concentration was determined by absorbance at 259 nm using ε = 15,400 M^−1^ cm^−1^. All experiments were performed in KMg50 buffer (10 mM imidazole, pH 7.0, 50 mM KCl, 1 mM MgCl_2_, 1 mM EGTA, 1 mM DTT) (5) unless otherwise noted.

### Expression plasmids, protein expression, and purification

Human MYO3A 2IQ WT without the kinase domain (amino acids 340–1143 of accession NM017433.4), was inserted into pFastBac with a C-terminal GFP tag and an N-terminal FLAG tag for purification (6) (Fig. 1*B*). A similar construct was generated using Quikchange site-directed mutagenesis (Agilent) that contained the H442N mutation. Protein was purified from SF9 cells *via* FLAG-affinity chromatography as previously described (6). After purification, myosin was incubated on ice with equimolar actin for 15 min, then spun in a Beckman TLA120.2 rotor at 95,000 rpm at 4 °C for 15 min. The resulting pellet was resuspended in KMg50 buffer, spiked with 2 mM ATP and an additional 150 mM KCl, and spun again to release the myosin from actin. The resulting supernatant was used for experiments (Fig. S2). Actin was purified from rabbit muscle acetone powder as previously described (63).

Human MYO3A WT lacking the kinase domain (amino acids 340–1616 of accession NM017433.4), was inserted into pEGFP to add an N-terminal GFP tag and allow for mammalian cell expression (6) (Fig. 1*B*). A similar construct was generated using site-directed mutagenesis that contained the H442N mutation. For motor swapped chimeras, we removed the MYO3A motor and first two IQ motifs (amino acids 340–1143 of accession NM017433.4) from the native construct by introducing a NOTI restriction site after the second IQ domain. We used PCR to amplify the various other myosin motors and first two IQ motifs (for primers, see Table S2) to ligate them into this MYO3A backbone. The cutoff for these amplifications was determined by alignment of the IQ motifs of each motor, ensuring that the length of the lever arm was consistent between the native MYO3A construct and the swapped in motors. The resulting constructs contained an N-terminal GFP, followed by the new myosin motors and 2IQ motifs (MYO1A, NMIIA, MYO5A, MYO7A, MYO10, MYO15; see Table S2 for accession # and exact amino acids) then finally the MYO3A tail (amino acids 1144–1616 of accession NM017433.4). The kinase dead (KD) construct contains the full sequence of MYO3A including the kinase domain (amino acids 1–1616 of accession NM017433.4), with the mutation K50R introduced into the kinase domain to abolish kinase activity (15, 26). Plasmids containing NMIIA, MY07A, and MYO15 were a gift from Dr Bechara Kachar.

### Steady state ATPase

The actin-activated ATPase activity of MYO3A 2IQ WT and H442N was examined using the NADH-coupled assay in KMg50 buffer at 25 °C as previously described (34, 64, 65). The rate of ATPase activity of 0.1 μM MYO3A 2IQ was measured using an Applied Photophysics stopped-flow over a 200 s period. Actin concentrations varied from 0 to 40 μM. The ATPase rates were plotted as a function of actin concentration and data were fit to a Michaelis–Menten equation (ATPase rate = *v*_0_ + (*k*_cat_∗[actin])/*K*_ATPase_ + [actin]), to determine the maximum rate of ATPase activity (*k*_cat_), the ATPase activity without actin (v_0_), and the actin concentration at which ATPase is one-half maximal ATPase (*K*_ATPase_). The ATPase activity without actin was determined with a higher concentration of MYO3A 2IQ (0.2–0.5 μM) to improve signal-to-noise.

### *In vitro* motility

The *in vitro* motility assay was performed at room temperature (22–23 °C) as previously described (6, 13). Briefly, MYO3A 2IQ WT or H442N was attached to a nitrocellulose coated coverslip *via* an anti-GFP antibody (Invitrogen) that bound to the C-terminal GFP tag. The surface was blocked with 1 mg/ml BSA in KMg50 to prevent non-specific interactions. AlexaFluor555-phalloidin-labeled F-actin was added to the flow cell before the addition of activation buffer. The activation buffer contained 0.35% methylcellulose, 10 μM calmodulin, 1 mg/ml BSA, 2 mM ATP, 46 units/ml pyruvate kinase, and 0.46 mM phosphoenolpyruvate all in KMg50 buffer. The addition of 5 mg/ml glucose and 0.1 mg/ml glucose oxidase catalase were included to reduce photobleaching. Finally, motility was observed using a Leica DMi8 TIRF microscope at room temperature (1 frame/10 s) and filament velocities were calculated in ImageJ using the MtrackJ plugin.

### Cell biology experiments

For live cell imaging experiments, COS7 or HeLa cells (ATCC) were trypsinized and plated at a density of about 40,000 cells on imaging dishes (35 mm Azer Scientific) for 24 h in DMEM supplemented with 10% fetal bovine serum at 37 °C and 5% CO_2_. After 24 h, cells were transfected with 2 μg of pEGFP constructs (WT, H442N, or motor swapped) using Lipofectamine 2000 transfection reagent (Invitrogen) and incubated for 24 h to allow overexpression. Cells were imaged in Opti-MEM media with no phenol red with a Leica DMi8 TIRF microscope equipped with a 37 °C stage objective warmer and 5% CO_2_ chamber at 2% laser power. Movies were generated by imaging every 10 s for 20 min. For all cells analyzed, protein expression was determined by average intensity of GFP fluorescence for each cell (Fig. S5). To measure extensions, filopodia that were clearly extended from the periphery of the cell and maintained within the focal place were tracked using the MtrackJ plugin. Filopodia extensions were only measured for filopodia extending within the focal plane for at least 60 s.

To determine filopodia length and tip localization, Leica LASX software was used to generate line scans for each filopodia as previously described (5). In short, for filopodia of length >1 μm, a line was drawn that extended from the filopodia tip to just within the cell body. The length of that line was used as the filopodia length. The fluorescence intensity of each line scan was then further normalized to the point with the highest intensity point for that filopodia, and the baseline was set by the linear fit to the fluorescence at the base. The tip-to-cell body (T/CB) ratio for each filopodia was determined by dividing the highest intensity point for a filopodia by the fluorescence at the base of the filopodia.

For fixed cell imaging, COS7 or HeLa cells were trypsinized and plated at a density of about 40,000 cells on glass coverslips in 6-well cell culture plates for 24 h in DMEM supplemented with 10% fetal bovine serum at 37 °C and 5% CO_2_. After 24 h, cells were transfected with 2 μg of pEGFP constructs (WT, H442N, or motor swapped) using Lipofectamine transfection reagent (Invitrogen) and incubated for 24 h to allow for overexpression. Cells were then fixed with 4% paraformaldehyde and stained with DAPI (nucleus) and AlexaFluor555 phalloidin (actin staining). Cells were then imaged after 24 h with a Leica SP8 confocal microscope.

### Inner ear hair cell experiments

Auditory hair cells were transfected with plasmid DNA encoding MYO3A KD or MYO3A KD H442N by the previously described injectoporation technique (66). Briefly, the sensory epithelium was dissected from C57BL/6 mice at postnatal day 5 in Hank’s Balanced Salt Solution (HBSS, Life Technologies) and the cochlear duct was opened by making an incision between Reissner’s membrane and the stria vascularis. The tissue was explanted by adhering it to a plastic, tissue-culture treated dish (USA Scientific) containing DMEM/F12 (Thermo Fisher Scientific) with 1 mg/ml penicillin. The culture was incubated at 37 °C with 5% CO_2_ for 2 h before injectoporation was performed. For the injection step, a glass micropipette with a 2 mm tip diameter loaded with plasmid DNA (2 mg/ml in water) was oriented perpendicular to the IHC row. The tip of the micropipette was inserted into the space between two IHCs and pressure was supplied by a microinjector to inject plasmid into the tissue. An ECM 830 electroporator was used to deliver a series of three 15 ms 60 V square-wave electrical pulses at 1 s intervals to platinum wire electrodes that were 2 mm apart and positioned directly over the injection site. After the electroporation, the culture media was exchanged with Neurobasal-A medium (ThermoFisher Scientific) supplemented with 2 mM L-glutamine (ThermoFisher Scientific), 1× N-2 supplement (ThermoFisher Scientific), 75 mg/ml D-glucose (ThermoFisher Scientific), and 1 mg/ml penicillin. Cultures were incubated for 18 h at 37 °C with 5% CO_2_, then fixed with 4% formaldehyde (Electron Microscopy Sciences) in phosphate-buffered saline (PBS) for 2 h and stained with Alexa Fluor 568 phalloidin (0.5 U/ml, Invitrogen) in PBS with 0.1% Triton X-100 (Millipore-Sigma) at room temperature for 1 h. The tectorial membrane was removed and the tissue was mounted in Prolong Diamond (Thermo Fisher Scientific). The cured slides were imaged with a Leica Plan Apo 63×/1.40 NA oil immersion objective on Leica SP8 inverted confocal microscope operating in resonant scanning mode (Leica Microsystems). Images were captured using Leica Application Suite X and deconvolved using Leica LIGHTNING deconvolution with the default settings. ImageJ was used to adjust display values for the images presented in figures.

### Statistical analysis

For kinetic experiments, unpaired Student’s t-tests were used to compare WT *versus* H442N. For cell biology and *in vitro* motility, SuperPlots (67) were created. Outliers were determined and removed *via* a Grubb’s test. Data was then compared with t-tests or ANOVA when appropriate. Linear regression was used for the correlation plot of *in vitro* motility velocity and filopodia extension velocity.

## Data availability

Data will be shared upon request to Christopher M. Yengo (cmy11@psu.edu).

## Supporting information

This article contains supporting information. Values measured in other studies are cited in Table S1.

## Conflict of interest

The authors declare no conflict of interest with the contents of this article.
