# Supplementary material for: The dynamics of actin protrusions can be controlled by tip-localized myosin motors
Source: J Biol Chem. 2023 Nov 30;300(1):105516. doi: 10.1016/j.jbc.2023.105516 (PMC10801316; doi:10.1016/j.jbc.2023.105516)
Supplement: Supporting Figures S1–S5 legends and Movies S1–S6 legends [file mmc1.docx]

**Supporting Information:**

**The dynamics of actin protrusions can be controlled by tip localized myosin motors**

Joseph A. Cirilo, Jr^1^, Xiayi Liao^2^, Benjamin J. Perrin^2^, and Christopher M. Yengo^1,3^

^1^Department of Cellular and Molecular Physiology, Penn State College of Medicine, Hershey, Pennsylvania 17033, USA.

^2^Department of Biology, Indiana University – Purdue University, Indianapolis, IN 46202, USA.

^3^Corresponding Author, cmy11@psu.edu

Figure S1: Density dependent *in vitro* motility. Velocities were measured for MYO3A 2IQ WT (A) or H442N (B) at different concentrations of myosin. Data are from 2 experiments. N=50 filaments per concentration, per construct.

Figure S2: Coomassie stained SDS-gel of FLAG-purified MYO3A 2IQ pre- and post-actin spindown. Lanes 1-7 are (1) molecular weight marker, (2) WT supernatant after actin spin down (final product used for experiments), (3) H442N supernatant after actin spin down (final product used for experiments), (4) WT pellet after actin spin down, (5) H442N pellet after actin spin down, (6) WT FLAG purified, (7) H442N FLAG purified, respectively. The bands for MYO3A 2IQ, actin, and calmodulin (CaM) are indicated.

Figure S3: Line scans of filopodia: Representative line scans of 10 filopodia for MYO3A WT (A) or chimeric myosins (B-D) demonstrating robust tip localization. For some protrusions, myosin localization events can be observed along the length of the filopodia, however, there is no trend between constructs.

Figure S4: Localization of chimeric myosin constructs in HeLa cells. Representative confocal images of fixed HeLa cells transfected with non tip-localizing chimeric myosins (A-F) and stained with Alexa Flour 555 phalloidin (actin) and Dapi (nucleus). Green is myosin, pink is actin, and blue is DAPI.

Figure S5: Average fluorescence intensity of each construct. The expression levels of each construct were estimated by examining the average fluorescence intensity of each cell that was examined. Data are from 3 separate transfections. There was no statistical difference between any transfection condition (ANOVA, p=0.2489). Error bars are ±SD.

Movie S1: Representative movies of MYO3A *in vitro* motility. MYO3A 2IQ WT (A) or H442N (B) was attached to a nitrocellulose coated coverslip via an anti-GFP antibody, before addition of fluorescently labeled actin and activation buffer, as described in the methods. Slides were imaged on a Leica DMi8 at 1 frame per 10 seconds.

Movie S2: Representative movie of MYO3A filopodia extension. COS7 cells were transfected with MYO3A WT and imaged on a Leica DMi8 scope, in TIRF at 1 frame per 10 seconds. Extensions were tracked and quantified using MtrackJ (representative track shown). Scale bar is 1µm.

Movie S3: Representative movie of MYO3A H442N filopodia extension. COS7 cells were transfected with MYO3A H442N and imaged on a Leica DMi8 scope, in TIRF at 1 frame per 10 seconds. Extensions were tracked and quantified using MtrackJ (representative track shown). Scale bar is 1µm.

Movie S4: Representative movie of MYO5.3A filopodia extension. COS7 cells were transfected with MYO5.3A and imaged on a Leica DMi8 scope, in TIRF at 1 frame per 10 seconds. Extensions were tracked and quantified using MtrackJ (representative track shown). Scale bar is 1µm.

Movie S5: Representative movie of MYO10.3A filopodia extension. COS7 cells were transfected with MYO10.3A and imaged on a Leica DMi8 scope, in TIRF at 1 frame per 10 seconds. Extensions were tracked and quantified using MtrackJ (representative track shown). Scale bar is 1µm.

Movie S6: Representative movie of MYO15.3A filopodia extension. COS7 cells were transfected with MYO15.3A and imaged on a Leica DMi8 scope, in TIRF at 1 frame per 10 seconds. Extensions were tracked and quantified using MtrackJ (representative track shown). Scale bar is 1µm.
