# Supplementary material for: The dynamics of actin protrusions can be controlled by tip-localized myosin motors
Source: J Biol Chem. 2023 Nov 30;300(1):105516. doi: 10.1016/j.jbc.2023.105516 (PMC10801316; doi:10.1016/j.jbc.2023.105516)
Supplement: Tables S1 and S2 [file mmc2.docx]

**Supporting Information:**

**The dynamics of actin protrusions can be controlled by tip localized myosin motors**

Joseph A. Cirilo, Jr^1^, Xiayi Liao^2^, Benjamin J. Perrin^2^, and Christopher M. Yengo^1,3^

^1^Department of Cellular and Molecular Physiology, Penn State College of Medicine, Hershey, Pennsylvania 17033, USA.

^2^Department of Biology, Indiana University – Purdue University, Indianapolis, IN 46202, USA.

^3^Corresponding Author, cmy11@psu.edu

Table S1: Motor properties of myosins used for chimeric constructs

|  | **Chimera** | **Sliding Velocity** | **Duty Ratio** |
| --- | --- | --- | --- |
| MYO3A | N/A | 70 nm/s * | 0.30 (5) |
| MYO7A | M7A.3ATail | 2.7 nm/s (49) | 0.60 (68) |
| MYO1A | M1A.3ATail | 60 nm/s (43) | <0.1 (47) |
| MYH9 | NMIIA.3ATail | 300 nm/s (46) | 0.05 (44) |
| MYO10 | M10.3ATail | **310 nm/s (28) | 0.60 (42) |
| MYO5A | M5A.3ATail | *290 nm/s (29) | 0.85 (45) |
| MYO15 | M15.3ATail | 429 nm/s (27) | 0.50 (27) |

References for each sliding velocity and duty ratio value are given in parentheses. *current study. **For MYO10 and MYO5A motility, value is from dimeric motors.

Table S2: Primers used in construct generation.

| **Amino Acids** | **mRNA Accession #** | **Primer Name** | **Sequence** |
| --- | --- | --- | --- |
| **NA** | **NM017433.4** | **huMYO3A H442N QC F** | 5’- ctggaaagactgaaaatgctaatcttttagttcagcagctga -3 |
|  |  | **huMYO3A H442N QC R** | 5’- tcagctgctgaactaaaagattagcattttcagtctttccag -3’ |
| **68-814** | **X67251.1** | **chMYO5A KPNI F** | 5’- gcgcggtaccgccgcctccgagctgtacacc -3’ |
|  |  | **chMYO5A NOTI R** | 5’- gaacgcggccgcccgcaggaatgtggcata -3’ |
| **2-788** | **U81453.1** | **muMYO7A KPNI F** | 5’- gcgcggtaccgttatcctgcagaagggggact-3’ |
|  |  | **muMYO7A NOTI R** | 5’- gaacgcggccgcctcatagtttttcctacagtggt -3’ |
| **2-787** | **U55042.1** | **boMYO10 KPN F** | 5’- gcgcggtaccgacaacttcttccccgagggaacac-3’ |
|  |  | **boMYO10 NOTI R** | 5’- gaacgcggccgcttttttcaggtgcaaaaatctcc -3’ |
| **1206-1938** | **AF144095.1** | **muMYO15 HINDIII F** | 5’- gcgcaagctttagatggagtggaggacatgacgca-3’ |
|  |  | **muMYO15 HINDIII R** | 5’-taccagcagatgaggcagagtctggcggccgcgttc-3’ |
| **2-772** | **AF127026.1** | **huMYO1A SALI F** | 5’- gcgcgtcgaccctctcctggaaggttctgtggggg-3’ |
|  |  | **huMYO1A NOTI R** | 5’-gaacgcggccgcggcagcctctgaccggaaatattt-3’ |
| **2-832** | **AJ312390.1** | **muNMIIA SALI F** | 5’- gcgcgtcgacgctcagcaggctgcagacaagtacc-3’ |
|  |  | **muNMIIA NOTI R** | 5’- gaacgcggccgcggtgaagagcctccaccactgcc-3’ |
