# Supplementary figures and images for: The dynamics of actin protrusions can be controlled by tip-localized myosin motors
Source: J Biol Chem. 2023 Nov 30;300(1):105516. doi: 10.1016/j.jbc.2023.105516 (PMC10801316; doi:10.1016/j.jbc.2023.105516)

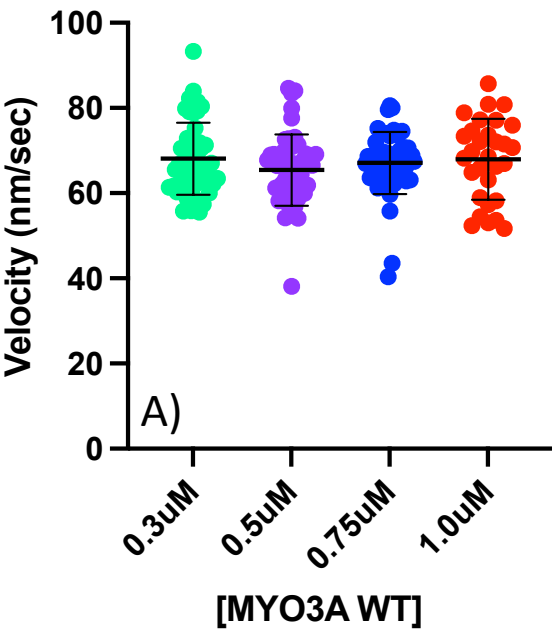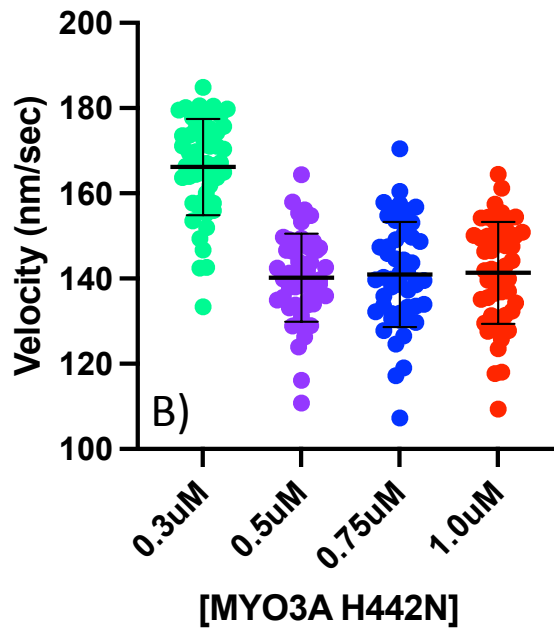

Supplement: Supporting Figure S1 [file mmc9.pdf]

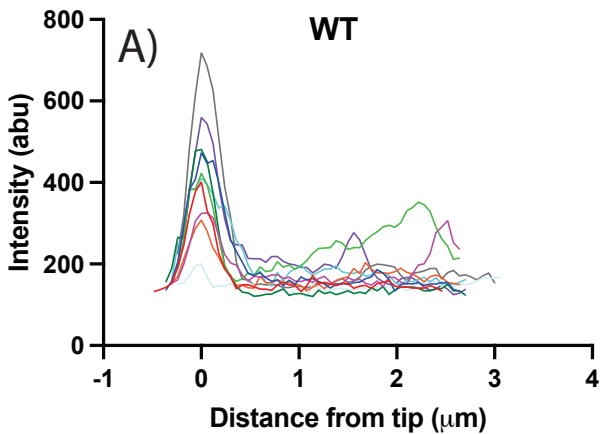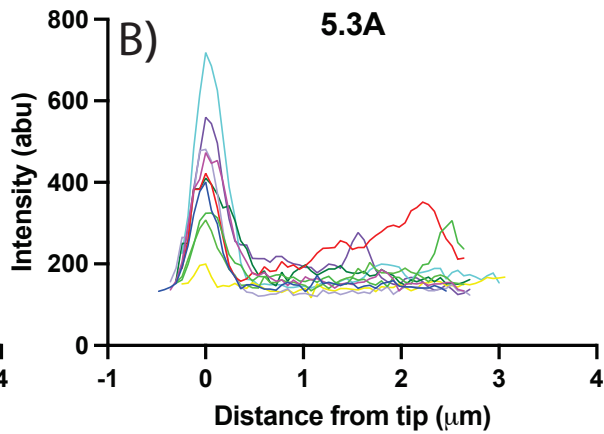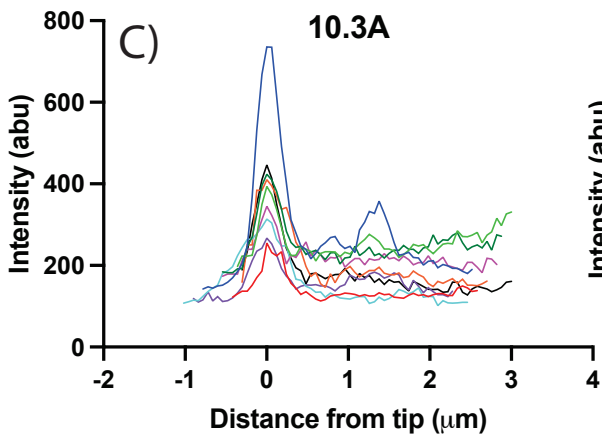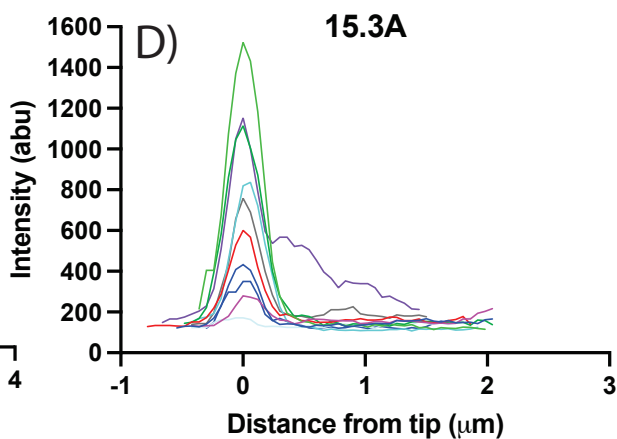

Supplement: Supporting Figure S3 [file mmc10.pdf]

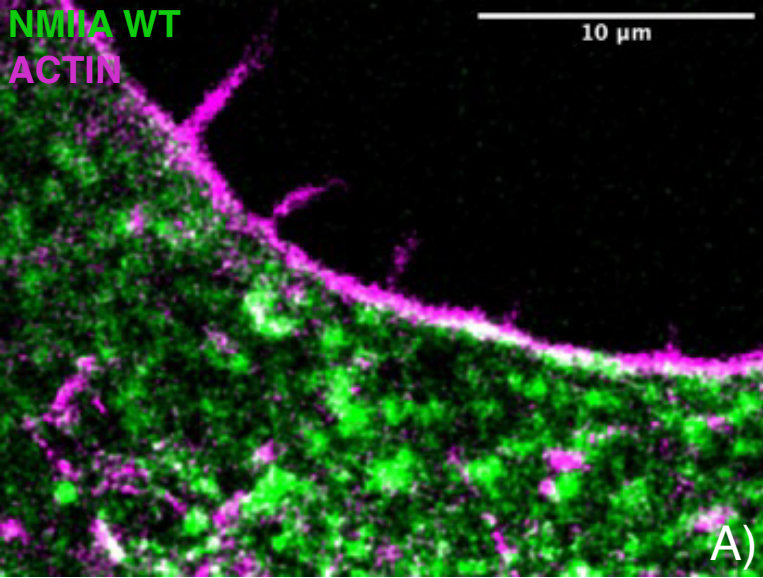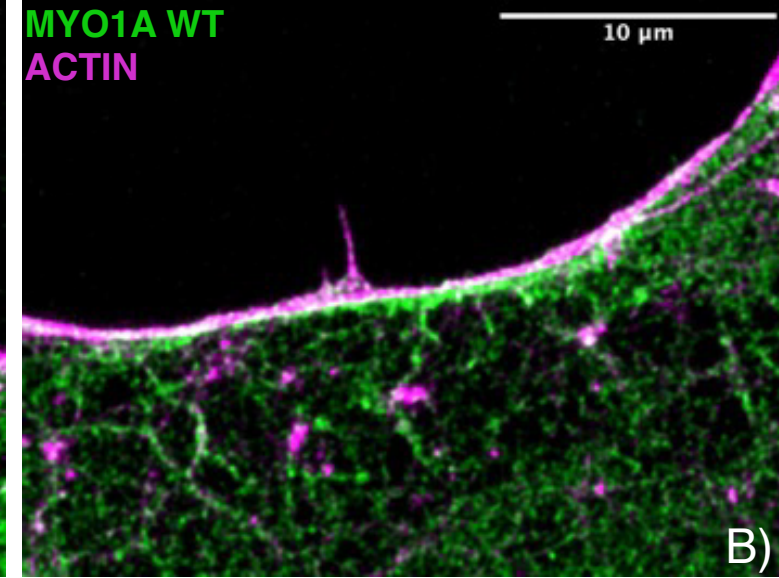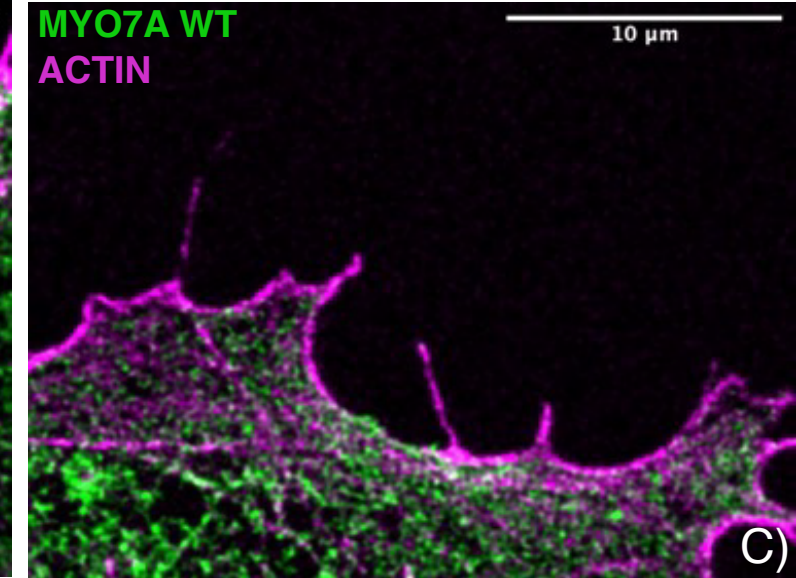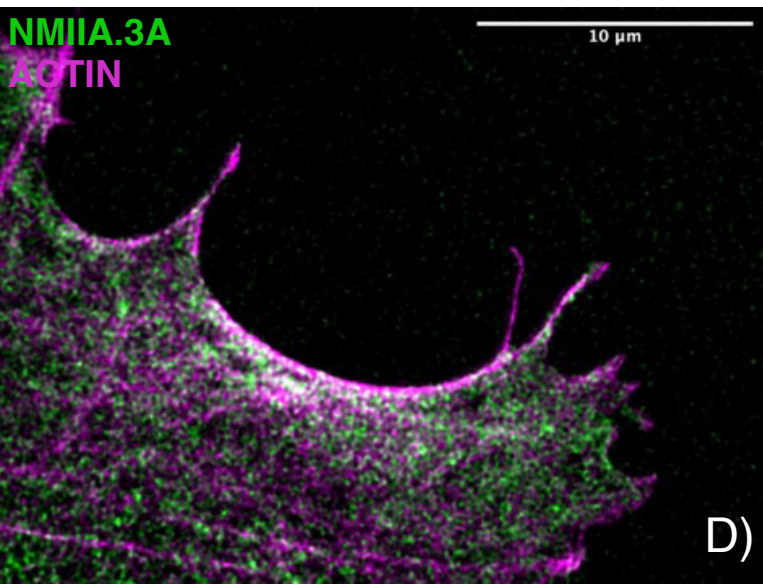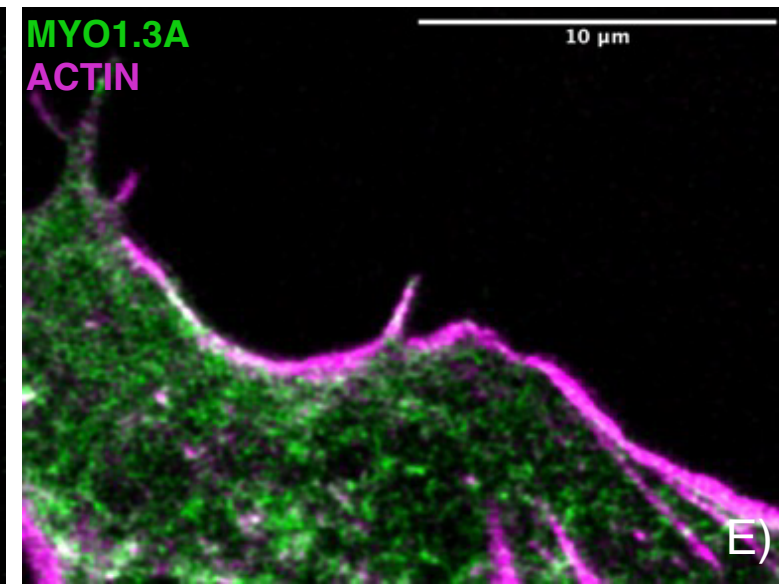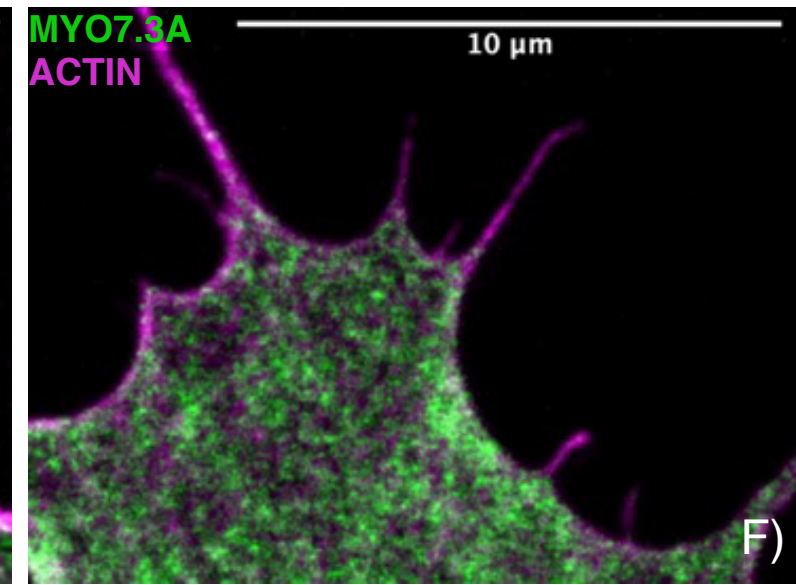

Supplement: Supporting Figure S4 [file mmc11.pdf]

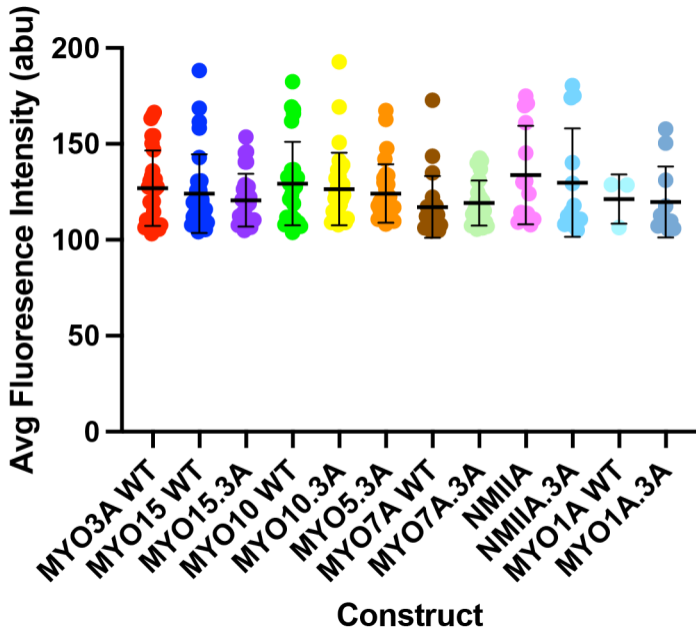

Supplement: Supporting Figure S5 [file mmc12.pdf]

**MW  
(kDa)**

**1 2 3 4 5 6 7**

**250**

**150**

**100**

**75**

**50**

**35**

**25**

**20**

**15**

**10**

**MYO3A 2IQ**

**Actin**

**CaM**

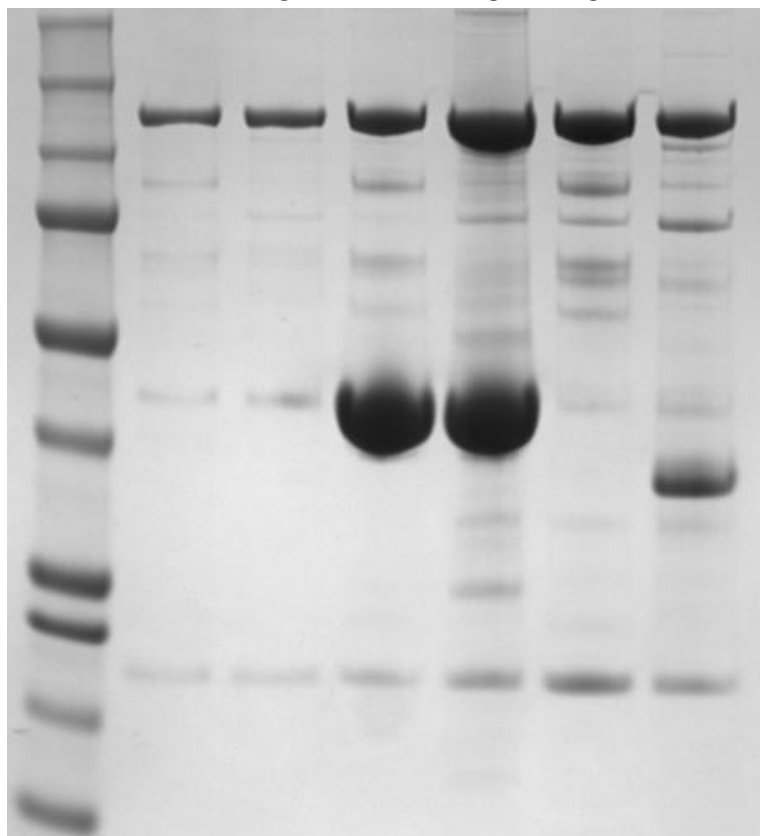

Supplement: Supporting Figure S2 [file mmc13.pdf]
